# Supplementary material for: Are carbon emissions trading and green financial instruments synergistic? -Comprehensive quantitative research based on content analysis
Source: PLoS One. 2024 Mar 7;19(3):e0298601. doi: 10.1371/journal.pone.0298601 (PMC10919601; doi:10.1371/journal.pone.0298601)
Supplement: S1 Data — (ZIP) [file pone.0298601.s001.zip › Data/地级市Z_F工作报告.zip!/║■─╧/╒┼╝╥╜τ╩╨/└┤╫╘╣1⁄2╓┌║┼í╛┬φ┐╦╩2╛▌═°í┐_2010.docx]

一、2009年及近三年工作回顾

　　2009年，是极不平凡的一年。2009年，是进入新世纪以来我国经济社会发展最为困难的一年。我们不仅经受了国际金融危机和甲型H1N1流感&ldquo;两股寒流&rdquo;的巨大冲击，而且局部地区还经历了五十年一遇特大旱情的严重挑战。但是，全市上下在市委的统一领导下，认真贯彻党中央、国务院、省委、省政府一系列方针政策，紧紧围绕建设世界旅游精品总目标，努力践行科学发展观，砥砺奋进，迎接挑战，共克时艰，奋力抢抓中央实施应对国际金融危机一揽子计划的重大机遇，奋力&ldquo;弯道超车&rdquo;，创新强基，乘势而上，强力推进&ldquo;三年有改观五年大变样&rdquo;进程，基本完成了市五届人大一次会议确定的各项任务，全面实现了&ldquo;保增长、保民生、保稳定&rdquo;的预期目标。

　　&mdash;&mdash;经济实力迈上新台阶。2009年，预计（下同）全市实现生产总值209亿元，比上年增长13.5%；实现财政总收入16.33亿元，其中一般预算收入12.04亿元，分别增长22.8%和26.4%；实现城镇居民人均可支配收入11620元、农民人均纯收入3180元，分别增长10%和8%。完成固定资产投资104.8亿元，比上年增长41.7%。完成重点工程投资42亿元，同比增长87.5%。争取中央新增项目投资3.99亿元，带动民间投资20亿元；引进省外境内资金24亿元、外资3026万美元，分别增长8.2%和31.2%；金融机构新增贷款45.3亿元，全行业盈利3.6亿元，分别增长40.4%和28.5%。实现社会消费品零售总额69.8亿元，增长18.5%；对外贸易逆势上扬，实现进出口总额2100万美元，增长1.37倍。区县经济各具特点，竞相发展，增长速度、质量和效益普遍提高。全市社会信心指数一路上扬，高达87%。

　　&mdash;&mdash;城乡基础设施建设快速推进，发展后劲不断增强。一年来，全市上下高水准、大力度推进基础设施项目建设，建成阳龙公路、张联公路、市中心汽车站和茶林河、长潭河水电站，完成核心景区公路改造，启动了荷花机场扩建二期工程，张花高速公路、张沅、慈石、桑鹤等干线公路和淋溪河水电站业已开工兴建，&ldquo;向上&rdquo;特高压线路建设、城乡电网改造快速推进，市城区基本实现3G网络覆盖，黔张常铁路和桑道、慈澧高速公路等项目列入国家或省建设规划。城市建设步伐加快，建成市城区鸬鹚湾路、武陵源高云路，大庸中路改造、仙人溪桥维修加固工程顺利竣工，土木溪水厂完成主体工程，陈家溪路、西溪坪路、八达路、天门山景区连接线、教场路和解放路延伸段建设接近尾声，贸易路、鸬鹚湾大桥、&ldquo;四路&rdquo;沿线环境综合整治&ldquo;穿衣戴帽&rdquo;工程和城市美化亮化工程建设步伐加快，贺龙国际体育中心、澧水风貌带建设正在征地拆迁。全市房地产业蓬勃发展，购销两旺，投资增幅达108%，排全省第一。与此同时，农村基础设施建设力度加大，建成通乡油路122公里、通村水泥路684公里、沼气池1.05万口，完成各项水利工程9220处，新修防渗渠道235.4公里，新增安全饮水人口10.25万人。按照耕地占补平衡原则，科学合理开发利用土地资源，在满足项目建设用地需求的同时，实现了城市土地收益4亿多元。

　　&mdash;&mdash;产业发展扎实推进，在挑战中取得新的成绩。创新营销，科学应对，旅游主导产业成功突围，接待规模再创历史新高。全市景区景点游客接待量达到1900多万人次，实现旅游总收入100.2亿元，均超过2007年水平，比上年分别增长15%和20%。旅游产业与文化产业加快融合，互促共进，国际乡村音乐周引起国内外媒体高度关注，中央领导肯定为&ldquo;巧用软实力提升国家形象的一次成功实践&rdquo;；&ldquo;天门狐仙&middot;新刘海砍樵&rdquo;填补了全省高端旅游文化节目空白。工业力克金融危机等负面影响，充分运用和采取中央、省里的一系列激活政策措施，快速成长。全市实现工业增加值38亿元，同比增长16%；其中规模工业增加值达到31亿元，实现利税2.7亿元，分别增长18%和20%。全市共完成工业园区建设投资5.6亿元，入园企业达到53家；完成工业技改投资10亿元，高新技术产业增加值占全部工业的比重达到10.3%。市县两级园区经济已具雏形，聚集吸纳能力明显增强。现代服务业发展空间不断拓展，势头良好。鸽子花酒店、&ldquo;十字街&rdquo;商务区投入运营，张家界体育生态公园、市博物馆、黄龙洞剧场、魅力湘西剧院以及一批五星级酒店正在抓紧建设。休闲、会展、商务等服务业日臻活跃，消费热点逐步增多。农业结构调整力度加大，产业化进程加快。全市实现农业增加值30亿元，同比增长5.5%，涌现了一大批城市农业、旅游农业、品牌农业和生态农业大户、小区和专业化村镇。现代烟草农业不断壮大，烟叶收购量达到24万担，实现利税2.6亿元，同比分别增长48%和64.5%。

　　在全面促进经济增长的同时，我市全面落实中央刺激消费政策，拉动了消费的增长，全市兑现财政补贴3200万元，大力实施家电、汽车、摩托车、农机下乡工程，直接拉动消费4.5亿元。

　　&mdash;&mdash;突出民本民生，各项社会事业全面发展。2009年32项省定为民办实事任务全面完成。市政府承诺的为农村已婚妇女免费普查妇女病等6件实事如期兑现。农民工返乡问题得到有效化解，新增城镇就业7850人，各类社会保险参保新增16万人，实现了城镇&ldquo;零就业&rdquo;家庭动态清零、城乡低保应保尽保。文教卫体环保等公共事业有了新的进步。教育&ldquo;普九&rdquo;负债基本化解，中小学校舍安全工程建设全面启动，吉首大学张家界学院一期工程如期竣工；公共卫生服务体系、重大疾病预防体系和医疗卫生应急机制不断完善，甲型流感疫情处于掌控之中；文化建设再创佳绩，荣获&ldquo;文化强省建设先进集体&rdquo;称号；农村广播电视&ldquo;村村通&rdquo;稳步推进，中心城区有线电视数字化转换基本完成；体育实现世界杯赛奖牌零的突破；人口和计划生育工作实现省定目标。生态建设和环境保护进一步加强。建成杨家溪和索溪峪（二期）污水处理厂，启动了慈利、桑植污水处理厂建设；完成营造林11.86万亩，扩大生态公益林补偿面积174.61万亩；万元GDP能耗下降4%，二氧化硫、化学需氧量排放总量分别削减5%和1.1%，城市空气质量优良率达到92.1%。安全生产全面加强，实现无重特大安全事故目标。

　　&mdash;&mdash;民主法制建设不断加强，社会和谐全面进步。多形式、多层面、全方位搭建民主平台，人民群众参政议政的积极性日益高涨。深入推进依法治市和全民普法，建立并不断完善法制教育体系、依法行政体系和法律监督体系，法制环境不断改善。大力开展&ldquo;平安满意在张家界&rdquo;专项活动，广大游客和市民对社会环境的满意度大幅提升。依法维护金融市场秩序，社会信用体系建设深入推进，处置非法集资初见成效。全市社会治安综合治理工作荣获全国最高荣誉&ldquo;长安杯&rdquo;，社会治安民意测评名列全省第三。社会应急管理和信访维稳工作不断加强，社会大局保持稳定。

一年来，全市上下同心同德，励精图治，各方面工作都顺利推进，监察、审计、统计、人事、编制、物价、国资监管、工商、税务、国土、质量监督、食品药品卫生监督、粮食、气象、外事侨务、民族、宗教、残联、移民、对台、档案、史志、国防、人防、无线电管理、新闻媒体等各项工作都取得了可喜的成绩。

　　各位代表，2009年，是我们实施&ldquo;三年有改观五年大变样&rdquo;建设的第三年。随着2009年全市经济社会发展的盘点与&ldquo;收官&rdquo;，我们可以豪迈地说：我市&ldquo;三年有改观&rdquo;的目标已经实现。三年的改观，不是单项的，也不只是城市面貌的日新月异，而是经济社会的全面进步。这三年，在省委、省政府的大力支持下，我们建成了一大批基础设施项目，拉动了我市经济社会的全面发展。各项数据说明，这三年是我市建市以来经济社会发展最快、最好的时期。三年来，我们实现了多项历史性突破：生产总值突破200亿元，三年净增81.5亿元，年均增长13.8%；人均GDP突破2000美元，达到13860元，净增5240元；全社会固定资产投资2009年当年突破百亿元，三年共完成投资242亿元，相当于前18年投资总和的三分之二。其中累计投入城市建设资金20多亿元，完成城市发展总体规划和土地利用总体规划修编，新建或改造20条主次干道，新增城市道路30公里、绿地面积138.5万平方米，新增城市建成区面积2.4平方公里，城镇化率由34%上升到38.6%。三年来，全市新增等级公路1069公里、发电装机42万千瓦、城市防洪堤21.5公里，农村油路通乡率、水泥路通村率分别达到95.4%和75.4%。张桑、阳龙公路的建成通车，构成了市区到区县的&ldquo;一小时交通圈&rdquo;；荷花机场改扩建一期工程、新火车站的建成使用，使全市面向国际国内的通道水准大幅提升。三年来，全市实现旅游总收入、第三产业增加值、城乡居民储蓄存款突破百亿元，三年分别净增20.8亿元、51.1亿元和50.3亿元；金融机构存款余额突破150亿元，三年净增66.1亿元；实际利用外资突破3000万美元，比2006年增长1.5倍；外贸进出口总额突破2000万美元，较2006年翻了两番；财政总收入突破15亿元、一般预算收入突破10亿元，连续三年保持20%增长，接近翻番；城镇居民人均可支配收入突破万元、农民人均纯收入突破3000元，年均分别增长11.2%和10%。

　　各位代表，总结三年来生动的建设实践，我们走过的道路并不平坦。在连续两年遭受多重冲击的严峻形势下取得这样的成绩，非常不易。这是党中央、国务院，省委、省政府正确领导的结果，是我们在市委统一领导下，认真贯彻市第五次党代会精神，扩大改革开放，不断创新完善发展思路和顶层设计的结果；是各级人大和政协大力支持、有效监督，全市各级政府一届接着一届干、一届力求比一届干得更好的结果，是全市各族人民奋力拼搏、砥砺前行、共克时艰的辉煌结果。在此，我代表市人民政府，向全市各族人民，向驻张部队、武警官兵和政法干警，向各民主党派、工商联、人民团体和各界人士，向所有关心支持张家界发展的各位新老领导和国内外朋友，表示诚挚的感谢，并致以崇高的敬意！

　　各位代表，&ldquo;三年有改观&rdquo;的成绩，不仅为我们实现&ldquo;五年大变样&rdquo;奠定了一个好的基础，也为我们加快推进世界旅游精品建设积累了许多宝贵的经验：

　　第一，&ldquo;发展是硬道理&rdquo;。只有坚持科学发展，代表先进生产力的发展要求，代表先进文化的发展方向，才能真正代表最广大人民群众的根本利益。张家界拥有世界一流的旅游资源，经过二十一年的奋斗，我们已经由一个旅游资源大市建设成为了一个旅游产业大市，但是基础差、底子薄、发展不快仍然是我们面临的主要矛盾。不管当前还是今后，放眼世界，立足市情，始终扭住第一要务，求真务实抓发展、干事业，这是我们的首要任务，任何时候都不能放松，任何时候都不能错位。只有学会发展，干出实绩，全市人民才会拥戴。

　　第二，解放思想，扩大开放，改革创新，是加快科学跨越发展的根本动力。&ldquo;为有源头活水来&rdquo;。没有思想的大解放，没有改革开放的大突破，就没有这三年的大改观。三年来，我们坚持在解放思想中不断完善顶层设计，确立了&ldquo;以旅游业为主导，新型工业强市，现代服务业兴市，加快城市建设，发展现代农业，促进扶贫攻坚&rdquo;的基本路子，并不断配套相关政策举措，使我们的路子越来越清晰；我们坚持在解放思想中创新体制机制，围绕破解发展瓶颈，相继实施投融资体制、城市建管体制、交通运输体制、电网管理体制、城乡绿化管理体制、土地管理体制等多项改革，制订出台了城市规划审批、征地拆迁补偿安置、项目建设行政性收费等一系列制度规范，市场体制开始理顺、机制逐步完善、活力不断释放；我们坚持在解放思想中推进对外开放，出台产业发展指导目录、鼓励和保护外来投资若干意见和招商项目落地责任制，突出引进战略投资者，开放引进的质量和效率不断提高，实际利用外资和省外境内资金分别是2007年前三年的1.76倍和1.72倍。

　　第三，突出改善民生，促进社会和谐，是科学统筹发展的历史责任。三年来，我们始终坚持把改善民本民生作为政府一切工作的出发点和落脚点，正确处理经济与社会、城市与农村、发展与环境的关系，努力推动和谐发展，得到了人民群众的拥护。全市各级财政共投入为民办实事建设资金18.3亿元，全面完成82项省定任务和12项市定任务；投入各类扶贫资金5.97亿元，减少农村贫困人口4.03万人；投入就业和社保资金17.2亿元，新增城镇就业2万人、农村转移就业9万人、各类社会保险参保58万余人，实现城镇职工和居民基本医疗保险全覆盖；投入保障性住房建设资金4.07亿元，建成廉租房2316套、经济适用住房3000余套；投入社会发展资金30.25亿元，实施了疾控中心、广电中心、博物馆等项目建设，建成义务教育合格学校42所、乡镇卫生院54所、乡镇文化站47个，农村广播电视覆盖率提高4.4个百分点，义务教育阶段学生全部实现免费就学，中小学D级危房全面消除；投入环保资金1.79亿元，城市污水、生活垃圾无害化处理率分别提高22.3和60个百分点，森林覆盖率、城市空气质量优良率分别提高1.7和5个百分点。

　　第四，以事业凝聚人心，用发展鼓舞人心，是加快经济社会发展的坚实基础。随着&ldquo;三年有改观&rdquo;建设的扎实推进，全市各族人民盼变化、求发展、干事业、图富强的信心指数和奋斗劲头空前高涨。三年来，通过实施&ldquo;四路&rdquo;综合改造工程，全市上下形成了团结拼搏、和谐奋进的&ldquo;四路&rdquo;精神；通过开展建市20周年庆典活动，认真总结从旅游资源大市到旅游产业大市跨越的辉煌业绩，全市上下形成了加快推进旅游大市向旅游经济强市迈进、实现第二次跨越的共同意志；通过深入学习实践科学发展观，落实党委、政府领导和部门联系重点项目、重点企业制度，全市上下形成了抢抓机遇，创新强基，强力推进&ldquo;三年有改观五年大变样&rdquo;进程的巨大合力。这正是我们基本实现&ldquo;三年有改观&rdquo;，奋力推进世界旅游精品建设的根本力量所在。

第五，科学决策，依法行政，民主办事，是提高人民政府公信力和执行力的重要保证。三年来，我们围绕构建开放、务实、诚信、效率、廉洁的服务性政府，不断完善定期向人大及其常委会、人民政协报告和通报工作情况制度，健全重大决策、重大事项征求人大代表、政协委员、工商联、各民主党派人士意见制度和社会公告、公示、听证制度；实行人大代表、政协委员、市民代表列席政府常务会议制度、重大决策征求专家意见制度、市长与市民对话制度；结合贯彻《湖南省行政程序规定》，修订完善了&ldquo;市人民政府工作规则&rdquo;，相继出台了&ldquo;政府重大行政决策程序规则&rdquo;、&ldquo;提高机关效能优化经济发展环境的意见&rdquo;和&ldquo;行政过错问责制度&rdquo;。这些制度的建立和落实，有效促进了政府执政水平和行政能力的提高。

　　各位代表，我们在总结成绩和经验的同时，也必须清醒地看到我们所面临的困难和问题。主要是基础差、底子薄、后发地区的基本市情还没有根本改变，上行的压力依然很大；交通等基础设施和城市功能与国际休闲度假旅游目的地的水准相距甚远，远不能适应产业发展对物质技术支撑平台的要求，打基础、强实力仍然是一项长期的主要任务；产业结构性矛盾随着发展的加快越来越突出，经济发展综合效益和水平不高，抗御风险的能力脆弱，自身发展的能力与加快发展的要求不相适应；城乡差距的矛盾还十分突出，部分群众生产生活还比较困难，缩小城乡差距还需经历一个长时期的努力，统筹城乡发展是我们面临的重要任务；发展的&ldquo;软环境&rdquo;还不宽松，市场化程度不高，体制、机制、结构性矛盾交织在一起，使我们应对和迎接国际国内产业转移的能力显弱；政府的执政能力和水平仍有待提高，解放思想依然是全市面临的重大命题；人均GDP迈过2000美元门槛，各种社会矛盾日渐凸显，社会治安和维稳的压力进一步加大，等等。这些问题，不同程度地制约着全市经济社会的科学跨越发展，我们必须直面正视，在今后的发展中不断加以解决。

　　二、关于2010年的工作

　　各位代表：经过三年的努力奋斗，我们已经站在了一个新的历史起点。2010年是实现&ldquo;十一五&rdquo;规划的最后一年，也是我们迈向&ldquo;五年大变样&rdquo;的关键一年。&ldquo;雄关漫道真如铁，而今迈步从头越。&rdquo;&ldquo;从头越&rdquo;，就是要不自满、不松劲，以更加高昂的斗志和更加务实的作风，超越&ldquo;三年有改观&rdquo;的既创业绩，努力实现&ldquo;五年大变样&rdquo;的跨越。

　　2010年总的发展环境将好于今年。国际金融市场渐趋稳定，主要经济体经济出现好转迹象，世界经济有望实现恢复性增长。国内经济回升向好，增速反弹态势强劲，增长基础逐步巩固，市场信心明显增强，2010年增速可望继续回升。扩大内需和改善民生的政策效应将继续显现，经济发展的动力、活力和竞争力在不断增强。从省内看，我省近几年通过&ldquo;一化三基&rdquo;战略实施，经济实现平稳较快发展，&ldquo;弯道超车&rdquo;势头强劲。当前我市正处于加速发展的大好时期。虽然我们仍面临着严峻的市场考验和竞争压力，国际国内还存在着许多不确定因素，从国际上看，世界经济复苏的基础并不稳固，欧、美、日等主要发达国家经济尽管已度过危难关头，但至今不能排除出现反复的可能，全球性挑战的压力仍然存在；从国内看，我国正处在经济社会发展的重要战略机遇期和社会矛盾的凸显期，经济运行中的新老矛盾相互交织，体制机制面临重大改革调整，保持经济社会平稳较快发展的任务十分艰巨；而我市由于仍处在底子薄、基础差、后发地区阶段，对外部环境的依存性大，抵御风险的能力仍然脆弱，强基础，调结构，上水平，不断提高我市的竞争力，仍将在相当一个时期内是我市经济社会发展的主要致力点。但是，中央继续实施积极的财政政策和适度宽松的货币政策，国家调整经济结构、转变发展方式的基本战略更加突出，扩大内需、刺激消费的政策导向更加明确，扶持&ldquo;三农&rdquo;、改善民本民生、促进中小企业发展、支持欠发达地区和生态环保地区的政策措施更加倾斜，特别是国务院出台《关于加快发展旅游业的意见》，把旅游业定位为国民经济的战略性支柱产业，无疑也为我们加快发展、乘势而上提供了极好的机遇。只要我们保持清醒头脑，科学判断形势，把准时代脉搏，善用政策机遇，顽强拼搏，继续创新强基，乘势而上，就能够为我们做好2010年各项工作，全面推进&ldquo;五年大变样&rdquo;工程实施争取更大主动。从而，争取更大的发展。

　　明年全市经济社会发展总要求是：全面贯彻党的十七大和十七届四中全会精神及中央、省委经济工作会议精神，以科学发展观为指导，紧紧围绕建设世界旅游精品总目标，继续解放思想，坚持扩大开放，以改革创新为动力，抓住机遇，踩准节奏，突出重点，&ldquo;强基础，调结构，重民生，促转变，上水平&rdquo;，奋力推进&ldquo;五年大变样&rdquo;工程的全面实施。

　　明年全市经济社会发展的主要预期目标是：生产总值增长13%以上，其中一、二、三产业分别增长5%、18%和15%；旅游接待人次增长10%，旅游收入增长12%；全部工业增加值增长17%，规模工业增加值增长19%；全社会固定资产投资增长25 %以上，社会消费品零售总额增长19%以上，外贸进出口总额增长10%；财政总收入增长15%以上，其中一般预算收入增长15%以上；城镇居民人均可支配收入增长10%，农民人均纯收入增长8%；居民消费价格指数涨幅控制在5%以内；新增城镇就业7500人，城镇登记失业率控制在4%以内；城镇化率达到40%；万元GDP能耗下降4%，主要污染物减排全面完成"十一五"计划，化学需氧量和二氧化硫分别控制在1.5和1.39万吨以内；人口自然增长率控制在9.3&permil;以内；亿元GDP生产安全事故死亡人数控制在0.6以内。

　　实现2010年经济社会发展的目标任务，全市上下要重点抓好以下十一项工作：

　　㈠认真贯彻落实中央和省委经济工作会议精神，进一步解放思想，紧密联系张家界的发展实际，加快转变发展方式。以大发展促大转变，以大转变促进更大的发展。2010年，全市经济社会发展必须遵循贯彻以下原则：一是致力推进经济结构调整，全面推进旅游经济由数量规模型向质量效益型转变，重点培育新型工业和现代服务业，大力发展现代农业，以产业结构转型升级带动经济发展质量和效益提升；二是致力推进城乡统筹，按照城乡一体化发展思路，突出中心城区建设，加快推进城镇化步伐，努力增强城镇综合承载能力，充分发挥城市对农村的辐射带动功能，围绕城市和旅游全面调整农业结构，实现"以城带乡"、促进新农村建设的目标；三是全面贯彻落实中央和省里的各项政策措施，全面激活市场，促进消费增长，以投资促消费，以消费促投资。四是致力推进科技强市，完善引导机制，加大扶持力度，全面促进产学研结合创新，认真实施国家知识产权战略，依靠科技进步推动经济结构转型升级，加快传统产业转型提质，发展绿色、低碳、循环经济；五是致力推进精细化管理，力促我市产业发展各项工作上水平。创新管理理念，健全管理制度，规范管理流程，强化管理机制，加快经济、社会和政务标准化管理体系建设，促进粗放型管理向精细化管理转变。六是把保障和改善民生作为保持经济平稳较快发展的重要增长点和促进社会和谐稳定的有效着力点，着力解决民生问题，始终加快发展社会事业，扩大和改善公共服务，不断提升人民的幸福指数。

㈡突出投资拉动，加快项目建设，带动全市经济平稳快速发展。一是强力推进事关我市全局发展的重大基础设施建设。重点抓好荷花机场扩建二期工程、张花高速公路、慈石、张沅、桑鹤等干线公路建设，力争黔张常铁路、环武陵源景区公路、张清公路城市段改造工程开工兴建，启动市城区绕城公路建设，实现天门山景区道路连接工程全面竣工和鸬鹚湾大桥新建工程主桥建成通车，加快实施市博物馆、张家界体育生态公园、贺龙国际体育中心和武陵源原生态绿景休闲园建设，继续推进城乡电网、城乡通信网络和城市防洪堤建设，力争实现重点工程建设投资68亿元、完成全社会固定资产投资135亿元。二是大力提高项目招商水平。优化政策，选准项目，紧紧抓住上海世博会、第五届中博会、湘台经贸周和"广交会"等重大机遇，全面扩大对外开放，在更大范围和更高层次引进战略投资者加入我市建设，促进我市发展，力争全年实际利用外资和省外境内资金增长10%以上。三是大力加强项目建设服务管理。优化环境，继续完善重大项目全程代理和绿色通道制度，全面落实重大项目市级领导、市直部门联系责任制和项目法人责任制、招投标制、工程监理制和合同管理制，确保各类项目顺利落地和开工建设。四是大力抓好项目库建设。2010年，我们将全面编制新一轮经济社会发展五年规划，即"十二五"规划。编制规划要以重大项目的调研、策划、论证和确立为重点，超前谋划一批事关长远的基础设施和产业转型项目，争取更多的项目进入国家和省"十二五"规划笼子，为全面推进我市由旅游产业大市向旅游经济强市跨越奠定扎实的基础。

　　　㈢围绕打造国际风景度假旅游城市，加快推进新型城市化。城市是现代经济发展的"火车头"。我市人均GDP达到了2000美元以上，已进入城市化快速成长时期。新型城市化是推动区域经济结构调整的重要依托，也是全面带动区域经济增长升级的引擎和动力。今后一个时期，我市要重点致力于强力推动中心城市的建设，同时要积极稳妥地加速以县城关镇为重点的小城镇建设，这是实现我市由旅游产业大市向旅游经济强市第二次大跨越的不争的战略选择。加速我市新型城市化建设，要紧紧抓住我市的特点，以优良的生态环境和特有的文化气质取胜，以"绿色、低碳、宜居"、功能齐全、综合配套、产业兴旺，打造区域性国际风景度假旅游城市品牌。2010年，要突出抓好三个方面的工作：第一，要依法全面宣传实施好新一轮城市总体规划，科学编制城市建设专业规划和区域性控制性详规，强力推进城市规划法定图则管理。进一步落实好城市功能布局和分区。要按照进一步做大做强做优做特的基本方针，加速中心城区的建设发展。按照整合、压缩、全面提升的原则，加速推进武陵源国际休闲度假区的建设。同时要按照新一轮城市体系规划，调整完善以县城关镇为重点的建制镇发展规划，积极推进我市各具产业发展特色和文化环境优势的小城镇建设，不断为我市经济社会强劲发展开辟新的战略空间。第二，要以设施配套，功能完善，科学合理为方针，不断提高城市的集聚能力和辐射能力为着力点，进一步加快城市基础设施建设。中心城区要以新城开发为重点，通过新城开发带动旧城设施和棚户区改造。2010年要全面完成中心城区贸易路、彭家巷路、西溪坪路、陈家溪路、八达路东段等建设工程，开工兴建溪北路、溪西路、中坪路等12条新城区道路。武陵源区要加快推进天子路、武陵路改扩建工程，同时要全面开工建设环景区公路。加快旧城改造续建项目建设进度，重点改造永定老城区北正街、天门路、南庄路和人民路，实施东门溪、西门溪治理。加快推进城市风貌特色建设，全面实施城市两区环境整治提升和"穿衣戴帽"工程，加快澧水风貌带建设。加快完善公用设施，消除中心城市无公厕街道。要加速慈利、桑植以县城为中心的配套建设，完成县城生活垃圾、生活污水处理设施建设工程，不断提高两县城的容纳空间和发展水平。第三，进一步加大城市管理力度。深化城市管理体制机制改革创新，提高依法管理城市水平。要严格规划管理，坚决制止违法违章建筑的滋长滋生，依法严厉处置违法违章建设，使我市城市建设发展步入健康有序的法制轨道。市容市貌管理要加速由粗放型向精细型管理转变，要创新机制，严格落实市容环境卫生门前"三包"责任制，不断加强城市交通、治安电子监控系统建设与管理，深入开展突出问题集中治理行动，实现城市秩序和市容市貌大改观。

　　　㈣创新营销，推动转型，不断增强旅游产业竞争实力。做强做优旅游产业，必须切实贯彻国务院关于加快旅游业发展的意见，统筹国内国际两个市场，在市场营销、产品创新和标准化服务、产业转型上实现新的突破。一是突出创意营销。加大资源整合力度，强化统一宣传措施，更加有效地推动城市形象和旅游产品的整体营销。加强文化元素挖掘与营造，全力促进文化产业的发展。以文化产业的发展促旅游，以旅游全面带动文化产业的发展。继续策划、创办具有轰动效应的主题营销活动，不断提升国际乡村音乐节品牌。重点支持大型山水实景音乐剧《天门狐仙&middot;新刘海砍樵》打造成湖南的名片。完善政策配套措施，不断扩大旅游航线和旅游专列，改革创新景区门票制度，增强市场开拓活力，全面搞活国内市场，有效拓展国际市场。二是突出新型产品培育。依托观光旅游资源，扎实推进休闲度假、商务会展、特色文化发展基础平台建设，加快实现我市旅游产品结构的调整转型，大力开发康体疗养、风情体验、攀登探险、生物地质科考等不同业态旅游产品。按照特色化、差异化发展原则，完善景区景点布局，统筹推进核心景区和东、西线旅游发展。深度研究、科学包装民族民俗和红色文化产品，做大做强文化旅游产业。三是要围绕开放、国际、世界旅游目的地，做好对外友好交往文章。通过国际友城关系，扩大张家界对外影响，不断开拓新的客源国。四是突出标准化服务。全面导入国际质量、环境和安全卫生标准，有效推进旅游服务和管理与国际接轨。要进一步加大力度引入大型旅游企业和中介商作为战略合作者进入我市，不断改造提升我市企业品质，不断提高我市竞争力。要以提高旅游经营单位和服务人员的诚信水平和服务质量，打造诚信旅游目的地为中心，继续深入开展"平安满意在张家界"活动，深入开展旅游环境专项整治行动，不断提高张家界美誉度和满意度。

㈤下大气力培植新型工业，加快推进我市新型工业化发展。推进新型工业化，是我市调整产业结构、增强经济实力的重点。要着力加快工业园建设步伐。进一步强化运行机制，加大政策扶持力度，加快基础设施建设，吸引产业、资金、技术向园区集聚。整合、优化市区工业园和慈利、桑植工业园产业布局，加快形成各具特色的园区经济。瞄准大企业、大集团，增强园区发展实力，力争到2010年底，全市工业园区新增入园企业15家。着力培育工业主导产业和产品。大力引导和扶持清洁能源、生物医药、新型材料、农产品加工、旅游商品、新型建材和汽车配件等优势产业发展，扩大产业规模，打造拳头产品。重点培育旅游商品产业龙头、打造重质碳酸钙产业链、加速五倍子系列产品开发、促进大鲵产业化发展。着力扶持骨干企业发展。加大招商引资力度，在引进战略投资和合作者上要实现新的突破。重点支持奥威科技、恒亮新材料、港越食品等规模骨干企业加快发展，力争年销售收入过亿元的企业达到20家。着力发展外向型经济，积极承接沿海地区产业转移，鼓励和培育企业"走出去，引进来"。着力推进企业体制、技术和管理创新，促进整体水平提高。帮助企业转变观念、创新体制机制，引导企业由 "家族经营"模式向现代企业管理模式转变，鼓励企业引进、开发科技项目和技术，培育人才队伍，提高科技素质。

　　　㈥深化农业结构调整，提高"三农"发展水平，加快构建具有张家界特色的现代农业产业体系。一是加快城市农业、旅游农业、生态农业和品牌农业发展。引导城市近郊村镇逐步退出传统种植品种，大力发展无公害蔬菜、健康养殖、名特优新水果、花卉苗木等高效产业。同时，要推进集约化和产业化发展。加强农产品产地、质量认证认定，培育一批具有地理标志特征的优质农产品品牌。积极发展观光农园和休闲农场，做优做强乡村旅游。二是加强现代农业基础设施建设。突出改善农村交通，争取实现100%的乡镇通油路，80%以上的村通水泥路。加强病险水库治理、防渗渠道改造和水土流失治理，新增10.25万安全饮水农村人口。继续实施农村低压电网改造、沼气池和信息网络建设，不断改善农村生产生活条件。三是加快农业产业化进程。扎实推进特色产业基地建设和标准化生产，加快发展现代烟草业，稳定烤烟20万担以上，新增蔬菜面积3万亩、优质茶园2万亩、时鲜水果5000亩、规模化养殖小区15个。扶持农业产业化龙头企业发展，加强农民合作组织建设，提高农业生产组织化、规模化水平。积极推广"公司+基地"、"协会+农户"、"农超对接"等经营模式，切实搞活农产品流通。四是加大扶贫攻坚力度，促进新农村建设。继续以桑植县为主战场，以产业开发为重点，全面推进整村扶贫工作，力争减少贫困人口1万人。实施农民工就业帮扶工程，稳定农村劳动力转移就业32万人。继续抓好新农村示范村片建设，力争35个省级示范村全部验收达标。

　　　㈦做大做强现代服务业，增强旅游经济活力。高起点推动生产性服务业发展。围绕构建湘西北地区金融创新示范区，加快发展现代金融业。围绕打造武陵经济区物流集散中心，加快开拓现代物流及配送业。围绕发挥航空口岸优势，加快探索临空经济产业。围绕争创国家再生资源回收体系建设试点城市，规范发展再生资源回收服务业。高水平改造提升消费性服务业。结合旧城改造和新区开发，以及小城镇建设，优化商业网点规划布局，着力培育一批上规模、高品位的特色商业企业和街区，提高我市旅游消费服务竞争力。加快发展社区居民服务业，启动"家政服务网络中心"等建设。大力发展具有旅游特色的房地产业，并将其培育成张家界经济社会发展新的支撑点。高质量培育新兴服务业。突出商务会展和休闲娱乐项目建设，推动休闲度假、商务会展、文化娱乐服务业快速成长。设立"会展办"，打造会展服务业品牌。完成"数字张家界"建设总体规划编制审定工作，启动网上办公等信息化应用项目建设。加快开发康体疗养、养老养生等特色服务业，不断拓展消费服务领域。高效率扩大城乡消费。加快农产品批发市场建设，启动粮食物流项目建设，推进城市社区标准化菜市场建设，继续抓好 "万村千乡市场工程"，深入开展家电下乡、汽车摩托车下乡和家电、汽车以旧换新活动，不断扩大消费热点。

　　　㈧大力改善民本民生，促进社会和谐发展。一是尽心竭力解决民生问题。继续把就业作为民生的头等大事来抓，切实做好城镇下岗失业人员、高校毕业生、城镇转业退伍军人和失地农民的就业工作，保持城镇零就业家庭动态清零。继续完善社会保障和社会救助体系，力争实现"应保尽保"和"需救即助"目标。开展农村社会养老保险试点。市人民政府继续集中财力物力，在全力实施省定为民办实事项目的同时，市政府决定再为群众办好5件实事，即：资助贫困大学生200名；建成市城区农产品市场质量检测实验室10个；改扩建乡镇敬老院27所，实现乡乡镇镇有敬老院目标；建成农村垃圾治理点253处；新建改建乡镇劳动保障站和社区劳动保障服务中心31个。二是全面推进社会事业进步。以加快教育强市建设为目标，加大保障力度，全面推进义务教育合格学校建设工程、中小学校舍安全工程和城区薄弱学校改造工程；积极发展学前教育，大力发展职业教育，全面提高高中教育质量，不断推进教育均衡发展；支持吉首大学张家界学院和张家界航院加快发展。高度重视科技人才队伍建设，努力增强科技创新能力，不断提高科技对经济社会发展的贡献率。以医药卫生体制改革为契机，健全完善以县级医院为龙头、乡镇卫生院和村卫生室为基础的农村医疗卫生服务体系，加快建设以社区卫生服务中心为主体的城市社区卫生服务网络，促进基本公共卫生服务逐步均等化。提高新型农村合作医疗保障水平和补助标准，力争参合率和补助标准分别达到90%和55%以上。推进文化体制改革，加快公益性文化设施建设和历史文物保护，加快城市社区、乡镇文化站、农村广播电视"村村通工程"建设。加强全民健身运动，精心筹办市第四届运动会，全力申办省第十二届运动会。坚持稳定低生育水平，统筹解决人口问题。加强未成年人保护和老龄工作，加强民族宗教、国防教育、人民武装和国家安全工作。三是加强民主法制和精神文明建设。积极创新和完善民主制度，进一步扩大社会各界和广大人民群众参政议政渠道，尊重民意，广集民智，促进发展。认真落实"依法治市"方略，全面提高社会法制化水平。充分发挥基层组织作用，以"守法纪、讲诚信、重礼貌"为主题广泛开展精神文明创建活动，扎实推进"文明张家界"建设。四是严格落实社会监管责任。严格国土资源管理，强化耕地保护责任，规范矿产资源开采行为。加强通胀预期管理和市场物价监管，确保价格总水平基本稳定。大力实施食品、药品、农产品放心工程，健全完善安全生产责任体系和应急处理体系，严防重特大安全事故发生。切实加大群众信访督办力度，及时有效化解社会矛盾。深入开展社会治安综合治理，严厉打击各种违法犯罪，确保群体性事件成功处置率达100%，确保色情敲诈、追客赶客等地域性案件100%查处到位，确保公众安全感指数和满意度明显提升。

㈨以创建绿色低碳宜居城市为重点，加快推进生态市建设。一是全面提升绿化水平。大力创建"森林城市"、"园林城市"，全面实施城市周边山地绿化美化工程，启动回龙观森林公园建设，加强城市公共绿地建设。进一步加强植树造林，力争新增营造林面积9万亩。严格保护世界自然遗产、世界地质公园和其他自然生态资源，力争实施核心景区新一轮移民搬迁工程。二是努力构建资源节约和环境友好型社会。坚持以低能耗、低污染、低排放为目标，转变发展方式，创新消费模式。大力推广使用清洁能源，全面执行建筑节能标准，不断戒除"一次性"消费用品。大力整治城区扬尘污染、城市废水排放、建设噪音和交通噪音。三是坚决落实节能减排任务。认真抓好工程减排、结构减排和管理减排，着力完善重点工业污染源、城镇污水集中处理设施监控体系，坚决淘汰落后产能和落后工艺项目，全面实现省定节能减排目标。四是切实控制农村面源污染。突出整治农村污水、畜牧养殖污染，全面启动农村垃圾治理"三年行动计划"，积极推进"五改"工程，不断改善农民生活居住环境。

　　　㈩深化体制机制创新，优化发展建设环境。体制机制创新，就是要剔除那些束缚生产力发展、存在于生产关系与上层建筑之中的落后东西，从而更好地解放和发展生产力。坚持以体制机制创新破解发展融资瓶颈。进一步推进社会信用体系建设，大力创建金融生态安全区，加强政银企对接，引导金融机构不断扩大信贷投放。建立健全金融服务体系，支持金融改革和产品创新，积极组建永定农村商业银行，支持发展担保机构和小额贷款公司，推进村镇银行建设，依法激活民间资本。支持符合条件的企业通过股份改造、发行债券和上市等方式，实现直接融资。科学整合现有融资平台资源，盘活各种存量优质资产，引进BOT、TOT等先进融资方式，努力破解城市建设、公用事业发展资金困难。坚持以体制机制创新改善投资和建设环境。继续深化行政审批制度改革，加快电子政务网络平台及电子监察等应用系统建设，积极推行行政许可项目在线审批。促进部门优质、高效和廉洁服务。扎实探索统一安置等有效途径，全力破解项目建设征地拆迁难题。不断规范、完善和落实招商引资政策措施，增强外来战略合作者投资兴业的信心和决心。坚持以体制机制创新规范市场秩序。深化市场监管体制、成品油价格体制、企业经营体制等重点领域的改革，抓紧出台旅游行业、商品质量、娱乐休闲场所、文化演艺场所、出租车、城市农贸市场等管理办法，形成科学合理、公平公正、行之有效的监管机制，打造规范有序、文明诚信、公众放心的市场形象。要进一步稳定和完善农村基本经营制度，推进土地适度规模经营和土地承包经营权流转，深化集体林权制度改革和农村综合改革，促进农业农村工作的更大发展。

　　（十一）依法行政，改进工作，切实提高政府执行力和公信力。实现明年经济社会发展目标，必须切实加强政府自身建设。一是认真实施政府机构改革，加快职能转变。按照省委、省政府的统一部署要求，整合部门职能，优化机构设置，把工作重点主要转到监管社会事务、服务市场主体、营造发展环境上来，把工作着力点主要放在保障和改善民生上来。二是坚持依法行政，提高行政水平。深入贯彻实施《湖南省行政程序规定》和《湖南省规范行政裁量权办法》，依法规范各种行政行为。全面落实行政问责和行政过错责任追究制度，强化行政管理。继续拓展政务公开渠道，主动接受人民代表大会及其常委会、人民政协、民主党派的监督和新闻舆论、社会公众的监督，确保权力始终在阳光下运行。切实加强信访工作，依法规范信访行为，畅通信访渠道，使信访真正成为人民政府联系人民群众的桥梁和纽带。三是严格政府绩效评估考核，提高工作效率。科学设置评估项目，完善考核措施，强化激励机制，坚决兑现奖惩，真正做到以实绩论奖惩，以发展论成败，在全市上下形成干实事、比实绩的生动局面。四是深入贯彻落实党的十七届四中全会精神，坚持从严治政。深入推进科学发展观学习实践活动，积极创建学习型机关，加强国家公职人员队伍素质建设，充分发挥党的先进性，努力提高执政能力。以行风政风评议为切入点，充分发挥行政监察等部门的职能作用，严厉查办行政不作为、缓作为、乱作为行为。深入开展商业贿赂专项治理和建设工程领域专项治理，重点完善国土出让、工程招标、产权交易、政府采购等制度，严肃查处各种腐败案件。切实加强廉政建设，认真落实厉行节约的各项制度规定，真正用好纳税人的每一分钱，努力建设人民满意的政府。

　　　各位代表："三年有改观"目标的实现，坚定了我们实现"五年大变样"的信心。让我们在省委、省政府和市委的正确领导下，再接再厉，锐意进取，为推进全市经济社会科学跨越发展而努力奋斗！
